# Antimicrobial activities and phytochemical properties of *Blumea balsamifera* against pathogenic microorganisms

**DOI:** 10.25122/jml-2021-0296

**Published:** 2022-08

**Authors:** Nurul Ain Ismail, Azlinah Matawali, Fadzilah Awang Kanak, Ping-Chin Lee, Siew-Eng How, Lucky Poh Wah Goh, Jualang Azlan Gansau

**Affiliations:** 1 Biotechnology Programme, Faculty of Science and Natural Resources, Universiti Malaysia Sabah, Sabah, Malaysia; 2 Preparatory Centre for Science and Technology, Universiti Malaysia Sabah, Sabah, Malaysia; 3 Biotechnology Research Institute, Universiti Malaysia Sabah, Sabah, Malaysia; 4 Industrial Chemistry Programme, Faculty of Science and Natural Resources, Universiti Malaysia Sabah, Sabah, Malaysia

**Keywords:** *Blumea balsamifera*, antimicrobial, phytochemical, alternative medicine

## Abstract

Medicinal plants have been widely used in healthcare based on traditional knowledge. We investigated the antimicrobial activities and phytochemical contents of a plant known as *Blumea balsamifera* (*B. balsamifera*), which Sabah native people have used for health benefits. Methanolic extracts and fractions of the leaves of *B. balsamifera* were tested for their phytochemical contents and their antimicrobial activities against four Gram-negative and five Gram-positive strains of bacteria. The extracts of *B. balsamifera* showed antimicrobial activities against three Gram-positive, and one Gram-negative bacteria, with the zone of inhibition ranging from 7.8 mm±0.41 to 10.5 mm±0.71. Fraction CE.F7 exerted the broadest antimicrobial activity towards four Gram-positive or Gram-negative bacteria. The phytochemical constituents identified in the extracts were alkaloid, flavonoid, steroid, and cardiac glycosides. The plant extract demonstrated antimicrobial activities and contained multiple phytochemical constituents. Further investigations into potential antimicrobial agents containing promising fractions would validate the medicinal properties of *B. balsamifera* used in Sabah.

## INTRODUCTION

Medicinal plants have been used in many different cultures throughout the world for their medicinal properties [1]. The acceptance and interest in natural therapeutics have surged greatly, to the point where they are currently available in a wide range of stores and markets. It is estimated that up to 80% of the developing regions rely on medicinal plants and traditional medical practice as a primary healthcare approach [2–4]. The usage of medicinal plants in the developed region of the UK and European countries has been fueled by the belief that it will promote healthier living and, consequently, the majority of the population uses medicinal plants as home remedies [5–7]. Despite the availability of modern pharmaceutical drugs on the market, natural products from medicinal plants are highly sought after for the bioprospecting of novel compounds as treatments for various diseases [8].

In Sabah, also known as North Borneo, the native people, such as the Rungus and Murut ethnics, have been using traditional medicinal plants with healing properties [9–11]. The practice of using medicinal plants for treatments is an essential heritage in the native people of Sabah. Sabah is widely known for its dense and high diversity in plant species, with over 5,000 species per 10,000 km^2^, out of which many are considered indigenous and are sparely used [12]. Underutilized plants used in folk medicine have recently been in the spotlight as scientists isolated pharmacologically active compounds and phytochemicals [13]. Hence, it is important to preserve the knowledge of medicinal plants in North Borneo. *Blumea balsamifera* (*B. balsamifera*), locally known as *Sambong, Telinga kerbau*, or *Tawawoh* is traditionally used to treat wounds and cuts, diarrhea, spams, infected wounds, respiratory infections, and stomach pains [14]. However, the biological properties of such a medicinal plant against pathogenic bacteria and their phytochemical contents remain understudied.

Currently, there has been a concern regarding the antimicrobial resistance of pathogenic bacteria against available antibiotics, which leads to a growing interest in harnessing the natural products of medicinal plants to discover novel drug candidates [15]. Plant-based antimicrobial agents are a vastly untapped source of medicines and have a large therapeutic potential [16]. They have been reported to treat infections and mitigate the side effects associated with synthetic antimicrobial and antibacterial drugs [17]. Therefore, the current study investigated the effects of *B. Balsamifera* for its antimicrobial activity against nine bacteria. The phytochemical contents were also correlated with the biological effects exerted by these plants.

## MATERIAL AND METHODS

### Sample collection and processing

*B. balsamifera* was collected from Kampung Seri Aman, Kota Marudu, Sabah, Malaysia. Voucher specimens were deposited at Borneosis gallery, Universiti Malaysia Sabah (Specimen No.: BORH 0970). Crude extracts were prepared in line with previously described procedures [18]. Briefly, dried leaves were homogenized to a fine powder. Approximately 500g of fine powder was extracted with methanol (1:10 w/v) for three days. The extract was filtered, concentrated under reduced pressure at 40℃, and stored at a lower temperature. The concentrated methanolic extract (ME) was further fractioned using liquid-liquid extraction to yield chloroform extract (CE) [19]. CE was further separated using column chromatography with silica gel (30 × 3 cm i.d, 0.040–0.063 mm, 230–400 mesh ASTM, Merck) with methanol: chloroform (1:19) solvent system and yielded 10 fractions (F1-F10) with 5 mL in each fraction.

### Antimicrobial screening

The antimicrobial screening was performed using a disc diffusion assay as described by Matawali *et al*., 2016 [20]. Gram-positive (*Bacillus cereus* (*B. cereus*), ATCC 11778, *Bacillus subtilis* (*B. subtilis*), ATCC 43223, *Staphylococcus aureus* (*S. aureus*), ATCC 25923, *Streptococcus pneumoniae* (*S. pneumoniae*), ATCC 6303) and Gram-negative (*Enterobacter aerogenes* (*E. aerogenes*), ATCC 13048, *Escherichia coli* (*E. coli*), ATCC 35218, *Proteus vulgaris* (*P. vulgaris*), ATCC 6380, *Pseudomonas aeruginosa* (*P. aeruginosa*), ATCC 9027, *Salmonella typhi* (*S. typhi*), ATCC 14028) bacteria were used in antimicrobial screening. Briefly, 20 µL of 1 mg/mL extracts/fractions dissolved in methanol were pipetted on a 6 mm Whatman filter paper disc and left to dry. Blank was used as a negative control. Then, the disk was placed on a nutrient agar plate inoculated with Gram-positive or Gram-negative bacteria strains. The plate was incubated at 37℃ overnight. Any zone of inhibitions (mm±SD) was measured using a Vernier caliper.

### Phytochemical screening of *B. Balsamifera extracts*

Extracts were analyzed qualitatively based on the presence of selected phytochemicals (alkaloid, flavonoid, saponin, steroid, terpenoid, tannin, phlobatannin, anthraquinone, and cardiac glycoside) according to standard methods [19, 21, 22].

## RESULTS

### Antimicrobial effects of extracts against several microorganisms

*B. balsamifera* extracts show antimicrobial activities against Gram-positive and Gram-negative bacteria (Table 1). Both ME and CE inhibited the growth of Gram-positive bacteria (*B. cereus, S. aureus*, and *S. pneumoniae*) with zones of inhibition ranging from 7.8 mm±0.41 to 10.5 mm±0.71. In contrast, ME and CE showed inhibitory activity against Gram-negative *P. aeruginosa* with a zone of inhibition of 7.5 mm±0.58 and 8.0 mm±0.82, respectively. Further antimicrobial screening of fractionated CE extracts (F1-F10) revealed that CE.F1, CE.F2, CE.F3, and CE.F7 exhibit antimicrobial activity. The broadest antimicrobial activity was shown by fraction CE.F7. However, no significant differences were observed when considering methanol extract as control.

**Table 1:** Antimicrobial activities (mean in mm±SD) from the extract obtained from *B. balsamifera*.

Microorganism	*B. balsamifera* (mm±SD.)					
	ME	CE	CE.F1	CE.F2	CE.F3	CE.F7
**Gram-positive**						
*Bacillus cereus*	9.0±1.41	10.5±0.71	0	6.5±0	6.5±0	6.5±0
*Bacillus subtilis*	0	0	0	0	0	0
*Staphylococcus aureus*	7.8±0.69	7.8±0.42	0	0	0	8.3±1.25
*Streptococcus pneumoniae*	7.8±0.41	8.1±0.49	0	0	7.4±0.75	7.9±1.32
**Gram-negative**						
*Enterobacter aerogenes*	0	0	0	0	0	0
*Escherichia coli*	0	0	0	0	0	0
*Proteus vulgaris*	0	0	0	0	0	0
*Pseodomonas aeruginosa*	7.5±0.58	8.0±0.82	7.6±0.48	0	8.8±0.96	8.0±0
*Salmonella typhi*	0	0	0	0	0	0

ME – Methanolic extract; CE – Chloroform extract; F1, F2, F3 & F7 – CC fractions from CE.

### Phytochemical screening

The phytochemical contents of *B. balsamifera* were summarized in Table 2. Flavonoid, steroid, and cardiac glycoside were present in both ME and CE. Fractions exhibited antimicrobial properties (CE.F1, CE.F2, CE.F3, and CE.F7) containing different levels of alkaloid, flavonoid, steroid, and cardiac glycoside. CE.F7, which exhibited the broadest antimicrobial activity, contained detectable levels of alkaloids, flavonoids, steroids, and cardiac glycosides.

**Table 2:** Phytochemical test result for *B. balsamifera* extract.

Test	B. balsamifera					
	ME	CE	CE.F1	CE.F2	CE.F3	CE.F7
**Alkaloid**	-	-	a	a	a	a
**Flavonoid**	a	a	-	+	+	+
**Saponin**	-	-	-	-	-	-
**Steroid**	+	+	+	+	-	+
**Terpenoid**	-	-	-	-	-	-
**Tannin**	+	+	-	-	-	-
**Phlobatannin**	-	-	-	-	-	-
**Anthraquinone**	-	-	-	-	-	-
**Cardiac glycoside**	+	+	+	+	+	+

a – moderately present; (+) – weekly present; (-) – No activity. ME – methanolic extract; CE – chloroform extract; CE.F1 – Fraction 1; CE.F2 – Fraction 2; CE.F3 – Fraction 3; CE.F7 – Fraction 7.

## DISCUSSION

Biomedical science has broadly explored plants as a potential source of novel drugs for human disease prevention and treatment. The search for plant extracts exhibiting antimicrobial activity is increasing due to the World Health Organization report indicating the rise of antimicrobial resistance [23, 24]. The present study revealed that the *B. balsamifera* fraction, CE.F7 exhibited the broadest antimicrobial properties against four bacteria tested, three Gram-positive (*B. cereus, S. aureus*, and S. penumoniae) and one Gram-negative (*P. aeruginosa*) bacteria. The difference in antimicrobial properties may be attributed to the difference in the bacterial cell wall structure. While Gram-positive bacteria possess a single layer in the cell wall, Gram-negative bacteria possess multiple layer structures and an outer cell membrane [25]. This was shown by previous studies, where herbal extracts from selected plants in Northern Iran have a stronger antimicrobial activity against Gram-positive bacteria than Gram-negative bacteria [26]. The ineffectiveness of the antimicrobial action against Gram-negative bacteria is due to their outer membrane. Major antimicrobial compounds must pass through the cell membrane through hydrophilic or hydrophobic interactions to reach their targets. However, the properties of the outer membrane of Gram-negative bacteria are selective and can be altered through genetic mutations and other factors, leading to resistance [27–29]. Hence, the discovery of novel antimicrobial compounds was important for future antibiotic security.

The differences in antimicrobial activity in the extracts and fractions were observed to be related to the differences in their phytochemical contents. Interestingly, the presence of flavonoids in CE.F2, CE.F4, and CE.F7 were correlated to the antimicrobial activity towards Gram-positive bacteria, in contrast to CE.F1, where no flavonoid was detected. This was supported by previous reports where there was a strong inverse proportional relationship between the levels of flavonoid content and the average minimum inhibitory concentration against Gram-positive bacteria [30]. The importance of phytochemical content has been recognized as an alternative to antibiotics, which could promote and enhance health [31]. The present study observed that CE.F7 has the broadest antimicrobial activity against Gram-positive and Gram-negative bacteria containing alkaloids, flavonoids, steroids, and cardiac glycosides. The richness of phytochemicals in medicinal plants is crucial to the antimicrobial spectrum [32]. Therefore, the extracts of *B. balsamifera* can be used as an antibacterial agent. Moreover, the fraction of CE.F7 warrants further investigation, and the exact mechanism of antibacterial effects needs to be examined for potential uses.

## CONCLUSION

Leaves of *B. balsamifera* contain antimicrobial properties against three Gram-positive bacteria (*B. cereus, S. aureus, S. pneumoniae*) and one Gram-negative bacteria (*P. aeruginosa*). The present study advocates for the need to investigate potential fractions that demonstrated antimicrobial activity, as future studies could lead to discovering new and effective antimicrobial agents.
